# Olaparib: A Clinically Applied PARP Inhibitor Protects from Experimental Crohn's Disease and Maintains Barrier Integrity by Improving Bioenergetics through Rescuing Glycolysis in Colonic Epithelial Cells

**DOI:** 10.1155/2021/7308897

**Published:** 2021-09-14

**Authors:** Dominika Kovács, Viola Bagóné Vántus, Eszter Vámos, Nikoletta Kálmán, Rudolf Schicho, Ferenc Gallyas, Balázs Radnai

**Affiliations:** ^1^Department of Biochemistry and Medical Chemistry, Medical School, University of Pécs, 7624 Pécs, Hungary; ^2^Division of Pharmacology, Otto Loewi Research Center, Medical University of Graz, Universitätsplatz 4, 8010 Graz, Austria; ^3^BioTechMed, 8010 Graz, Austria; ^4^Szentagothai Research Centre, University of Pecs, 7624 Pecs, Hungary; ^5^HAS-UP Nuclear-Mitochondrial Interactions Research Group, 1245 Budapest, Hungary

## Abstract

Crohn's disease (CD) is an inflammatory disorder of the intestines characterized by epithelial barrier dysfunction and mucosal damage. The activity of poly(ADP-ribose) polymerase-1 (PARP-1) is deeply involved in the pathomechanism of inflammation since it leads to energy depletion and mitochondrial failure in cells. Focusing on the epithelial barrier integrity and bioenergetics of epithelial cells, we investigated whether the clinically applied PARP inhibitor olaparib might improve experimental CD. We used the oral PARP inhibitor olaparib in the 2,4,6-trinitrobenzene sulfonic acid- (TNBS-) induced mouse colitis model. Inflammatory scoring, cytokine levels, colon histology, hematological analysis, and intestinal permeability were studied. Caco-2 monolayer culture was utilized as an epithelial barrier model, on which we used qPCR and light microscopy imaging, and measured impedance-based barrier integrity, FITC-dextran permeability, apoptosis, mitochondrial oxygen consumption rate, and extracellular acidification rate. Olaparib reduced the inflammation score, the concentration of IL-1*β* and IL-6, enhanced the level of IL-10, and decreased the intestinal permeability in TNBS-colitis. Blood cell ratios, such as lymphocyte to monocyte ratio, platelet to lymphocyte ratio, and neutrophil to lymphocyte ratio were improved. In H_2_O_2_-treated Caco-2 monolayer, olaparib decreased morphological changes, barrier permeability, and preserved barrier integrity. In oxidative stress, olaparib enhanced glycolysis (extracellular acidification rate), and it improved mitochondrial function (mitochondrial coupling efficiency, maximal respiration, and spare respiratory capacity) in epithelial cells. Olaparib, a PARP inhibitor used in human cancer therapy, improved experimental CD and protected intestinal barrier integrity by preventing its energetic collapse; therefore, it could be repurposed for the therapy of Crohn's disease.

## 1. Introduction

Inflammatory bowel disease (IBD) is a chronic and remitting inflammatory disease of the gut. More than 1 million inhabitants in the USA and approximately 2.5 million in Europe suffer from IBD, and its incidence is permanently rising [1]. IBD exhibits two main forms, namely, ulcerative colitis (UC) and Crohn's disease (CD), and it appears in flare-up and remission phases [2]. Although UC and CD are two distinct forms of IBD, they share the phenomenon of epithelial barrier dysfunction. Barrier failure often results in increased intestinal permeability, a condition called “leaky gut” [3]. In this disorder, the gut microbiota can directly enter the colonic tissue and induce the activation of immune cells causing chronic inflammation [4, 5]. The initiators of increased gut permeability are not clearly elucidated, but it is often suggested that increased permeability is a consequence of altered energy metabolism and mitochondrial dysfunction of intestinal epithelial cells (IEC) [6]. For example, investigations with conplastic mouse strains, which share the same nuclear genome but have different mitochondrial genomes, demonstrated that those mice with high mucosal respiratory chain activity and elevated concentration of ATP develop less intense colitis than those that produce a smaller amount of mucosal ATP [7]. In CD patients, increased mucosal permeability in the ileum was accompanied by mitochondrial swelling and decreased ATP concentration [8]. In addition, the activity of complex II (CII), a part of the mitochondrial electron transport chain (ETC), was found to be abolished in the colon of UC patients [9]. Another group found lower levels of CI and CIV in IBD patients compared to control subjects and also measured lower ATP concentrations [10]. Furthermore, enhanced lactate levels were found in CD patients in comparison with healthy individuals, which correlated with the disease activity [11]. All these results suggest mitochondrial dysfunction, disturbed oxidative phosphorylation, and enhanced glycolytic activity in the mucosa of IBD patients.

Under physiological conditions, IECs use butyrate as a primary energy source [12]. Butyrate is produced by several species of the microbiota, and it is catabolized in IECs via *β*-oxidation and citric acid cycle (CAC) [13–15]. In addition, dehydrogenases of these catabolic pathways reduce NAD^+^ and FAD to NADH+H^+^ and FADH_2_ which promote the reduction of the mitochondrial respiratory chain CI and CII [16]. Thereafter, CI, CIII, and CIV pump protons across the inner membrane from the matrix to the intermembrane space raising a proton gradient [16]. At the end of ETC, CIV consumes O_2_ and reduces it to H_2_O. Finally, the proton gradient drives F_O_F_1_-ATPase, which produces ATP from ADP and P_i_ [16].

However, in inflammation, mitochondrial dysfunction and mitochondria-derived ROS increase. Under these circumstances, IECs switch their metabolism from oxidative phosphorylation (OXPHOS) to aerobic glycolysis [13, 17]. In aerobic glycolysis, glucose transforms to lactate without oxygen consumption, although sufficient amount of oxygen is present in the cells [18]. In this situation, glycolysis produces ATP and, as a by-product, lactate is synthesized from pyruvate by lactate dehydrogenase [17]. Since the mitochondria are a major source of ROS [19], the catabolic pathway via glycolysis and lactate dehydrogenase bypasses the mitochondria and do not feed mitochondrial ROS generation [20]. Thus, the cell shuts down the mitochondria to protect itself from mitochondrial ROS [21]. This concept is strengthened by the findings that proinflammatory cytokines (TNF-*α*, IL-1*β*, and IFN-*γ*) increased the rate of glycolysis in rat enterocytes and also triggered ATP turnover [22]. Also *C. rodentium* infection in mice induced aerobic glycolysis and enhanced the level of sodium-glucose transporter 4 and lactate dehydrogenase A. At the same time, enzymes of CAC and OXPHOS were downregulated [23]. Most importantly, a strong expression of glycolytic enzymes was found in the colon of IBD patients [24]. In active CD, lactate levels were significantly higher compared to the control subjects [11]. Therefore, in colitis, aerobic glycolysis becomes the main source of ATP. Nevertheless, in severe inflammation, activation of the enzyme poly(ADP-ribose)-polymerase-1 (PARP-1) blocks glycolysis [25], i.e., it terminates the “last safe way” of energy production and forces the cells along the death pathway causing strong mucosal damage with severe ulceration and compromised barrier function.

PARP-1 has been long involved in cancer development and inflammation. Accordingly, PARP-1^−/−^ mice were protected in 2,4,6-trinitrobenzene sulphonic acid- (TNBS-) induced colitis [26] and pharmacological inhibitors of PARP-1 improved dextran sodium sulfate-induced [27] and TNBS-induced colitis [28] in rodents. PARP-1 is activated by DNA damage and catalyzes polyADP-ribosylation (PARylation) of numerous nuclear proteins using NAD^+^ as a substrate [29]. This process is a part of the DNA damage response leading to activation of the DNA repair enzymes [30]. However, excessive PARP activation can totally deplete NAD^+^ pools, which makes cellular energy metabolism impossible [31]. Several lines of evidence demonstrate that PARP activation not only depletes NAD^+^ pools but also inhibits the enzyme hexokinase, which catalyzes the first step of glycolysis [25]. As a result, repressed glycolysis cannot feed CAC with Acetyl-CoA (produced by pyruvate dehydrogenase from the glycolytic end-product pyruvate), and CAC is not able to reduce NAD^+^ and FAD to feed mitochondrial ETC and OXPHOS [32], so PARP-induced mitochondrial dysfunction originates, at least partially, from the decreased substrate flow from glycolysis to CAC and ETC [25]. Since, in severe colitis, glycolysis is the main source of ATP (because of mitochondrial shutdown) [21] and also glycolysis is inhibited by PARP [25], IECs have to face with energetic collapse and they lose the ability to form a strong and continuous barrier [7].

In the present study, we investigated whether olaparib, a PARP inhibitor used in human cancer therapy, has a beneficial effect in a CD mouse model and, accordingly, whether it could be repurposed for CD treatment. To answer this question, we applied olaparib during a TNBS-induced experimental colitis model. Additionally, since IECs are the first line of defence in the colon and barrier interruption is a hallmark of IBD, we used Caco-2 colonic epithelial cells and investigated barrier function and energy production *in vitro*.

## 2. Materials and Methods

### 2.1. Animals and Experimental Colitis

**Figure 1:**
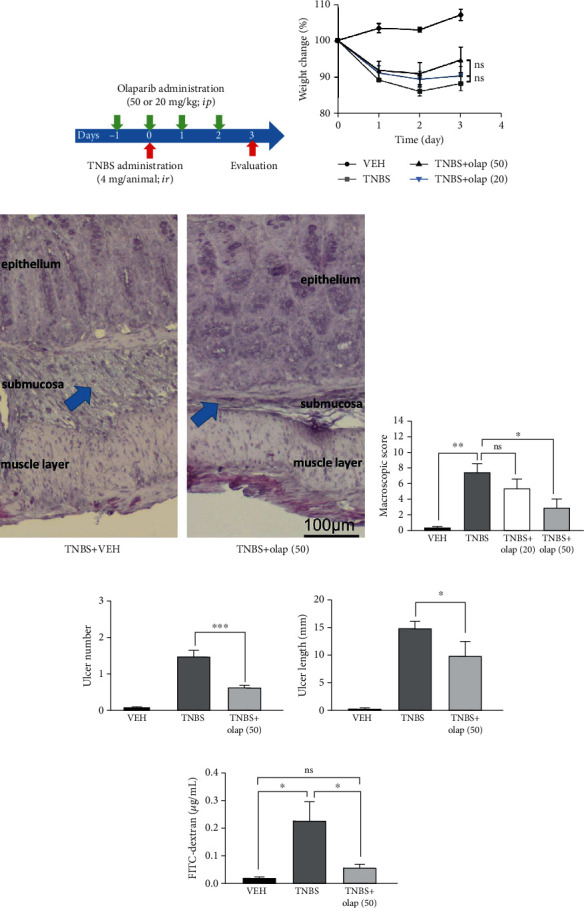
Olaparib treatment attenuated TNBS-induced colitis in mice. (a) Experimental protocol of TNBS-induced colitis and olaparib treatment. (b) Bodyweight changes (percentage of the initial bodyweight of each animal) in every experimental group. Data from one of three independent experiments are expressed as mean ± SEM (*n* = 4-7). (c) Representative images of hematoxylin staining of colon cross-sections from the TNBS- and TNBS+olap (50)-treated mice. Arrows indicate the most affected part of the colon tissue, the submucosa. (d) Macroscopic score in every experimental group. Data combined from 2 separate experiments (*n* = 9-21). (e) Ulcer number and (f) ulcer length in the VEH, TNBS, and TNBS+olap (50) groups. Data combined from 3 separate experiments (*n* = 9-27). (g) Intestinal permeability based on the measurement of FITC-dextran in blood samples 3 days after TNBS treatment. Data from one of three independent experiments are expressed as mean ± SEM (*n* = 5-8); ns: not significant; ^∗^*P* < 0.05, ^∗∗^*P* < 0.01, and ^∗∗∗^*P* < 0.001. VEH: vehicle; TNBS: 2,4,6-trinitrobenzene sulphonic acid; olap (50): 50 mg/kg olaparib; olap (20): 20 mg/kg olaparib.

Male CD1 mice (Jackson Laboratory, Bar Harbor, ME, USA) were bred and maintained at the SPF animal facility of the Department of Immunology and Biotechnology, Medical School, University of Pécs. At the age of 6-8 weeks, they were transported to our animal house facility and acclimatized for 2 weeks under standardized circumstances. Standard laboratory chow and water were available ad libitum. Experimental procedures were approved by the Animal Research Review Committee of the University of Pécs, Medical School (Permit number: BA02/2000-4/2017). For the colitis experiments, we used the vehicle (VEH), TNBS, and TNBS+olaparib (TNBS+olap) treatment groups. In total, 72 animals were used; 1 mouse died during the experiments before evaluation. In our experimental setting, every group contained 3-9 animals. We performed 3 independent experiments including in total 9 VEH, 26 TNBS, 9 TNBS+20 mg olaparib, and 27 TNBS+50 mg olaparib). The age-matched (8-10 weeks), sex-matched (male), and bodyweight-matched (30-40 g) animals were randomly divided into groups by a technician. During the experiments, mice were individually housed to avoid aggressive behavior. Individual housing was approved by the Animal Research Review Committee of the University of Pécs. The experimental period lasted in total for 4 days (Figure 1(a)). On day 1, olaparib treatment started (pretreatment), and thereafter, we administered it daily once for 3 times (thus, in total, we performed 4 olaparib treatments). On day 0, animals were treated with TNBS (1 bolus), and on day 3, mice were anesthetized and euthanized. Olaparib (AZD2281, MedChemExpress, New Jersey, USA) was administered intraperitoneally (single injection) on the day before TNBS challenge, followed by daily administration for 3 days at the dose of 20 or 50 mg/kg bodyweight. The applied dose of olaparib was selected based on literature data [33]. The vehicle group received sterile distilled water containing 4% DMSO and 30% PEG300. After 12 hrs fasting, mice were anesthetized with 5% isoflurane (Baxter Hungary Ltd., Budapest, Hungary) in 100% oxygen in an anaesthetic chamber. Colitis was induced by a single intracolonic injection of TNBS (4 mg in 100 *μ*l of 30% ethanol; Sigma-Aldrich, Missouri, USA) through a catheter inserted 3 cm into the colon. The VEH group received an equal volume of 30% ethanol. Animals were weighed daily during the experiment and sacrificed 72 hrs after TNBS administration. Mice were anesthetized with 5% isoflurane and decapitated gently by a dedicated surgical scissor to collect the highest possible amounts of trunk blood. This technique was approved by the Animal Research Review Committee of the University of Pécs. Trunk blood was collected; the colons were removed, measured, weighted, and opened longitudinally to detect the macroscopic colon damage. Tissue samples were processed for further analyses. Treatments and macroscopical scoring were carried out blind.

### 2.2. Intestinal Permeability Measurement

Intestinal permeability was determined by measuring the concentration of fluorescein isothiocyanate (FITC)-dextran (40 kDa; Sigma-Aldrich Missouri, USA) in serum. 3 days after TNBS treatment, FITC-dextran solution (100 *μ*l of a 60 mg/ml solution) was administered intrarectally. Serum was collected 1 hour after the administration, and fluorescence intensities were detected by a Promega GloMax plate reader (excitation, 490 nm; emission, 510–570 nm). A standard curve was generated from a serial dilution of FITC-dextran in PBS.

### 2.3. Hematological Analysis

At the endpoint of the TNBS model, mice were anesthetized with 5% isoflurane and decapitated gently by a dedicated surgical scissor, and trunk blood was collected directly into microtainer tubes (Becton Dickinson, Hungary) containing EDTA as an anticoagulant. Hematological parameters were determined by a Sysmex XN-1000-V Multispecies Hematology Analyzer (Sysmex America Inc., USA) within 2 hours of sampling. Lymphocyte to monocyte ratio (LMR), platelet to lymphocyte ratio (PLR), neutrophil to lymphocyte ratio (NLR), and neutrophil to monocyte ratio (NMR) were calculated from the absolute cell counts for each animal separately.

### 2.4. Macroscopic Scoring

Colonic tissue damage score was assessed by a macroscopic scoring system described previously [34]. Briefly, individual points were added for ulcers (0.5 points for each 0.5 cm), adhesions (0 points = absent, 1 point = 1 adhesion, and 2 points = 2 or more adhesions or adhesions to organs), colon shortening, based on a mean length of a healthy colon (1 point = >15%, 2 points = >25%), wall thickness (measured in mm), consistency of the stool, and the presence of blood in the stool (hemorrhage, fecal blood, or diarrhea increase the total points by 1).

### 2.5. Histology of Colon Tissue

Segments of the distal colon were stapled flat onto a cardboard with the mucosal side up and fixed for at least 24 hrs in 10% neutral-buffered formalin. Tissue was then dehydrated and embedded in paraffin, and standard hematoxylin staining was performed on 5 *μ*m thick sections. To this end, slides were deparaffinized, cleared in xylol, rehydrated in a descending ethanol series, stained with hematoxylin solution according to Gill II, and cleared in tap water. Images were taken with an Olympus DP50 camera and processed with cellSens imaging software (Olympus, Vienna, Austria).

### 2.6. Cytokine Levels of Colon Tissue

Levels of inflammatory cytokines IL-1*β*, IL-6, TNF-*α*, and IL-10 were measured in colon tissues. Tissue was homogenized mechanically in an extraction buffer supplemented with protease inhibitor cocktail (Sigma-Aldrich, Missouri, USA). Bradford assay (Bio-Rad Laboratories, California, USA) was used to measure the concentration of total protein. Subsequently, normalization of protein concentrations was performed and cytokine levels were determined by Ready-Set-Go ELISA kits (eBioscience, California, USA) according to the manufacturer's instructions.

### 2.7. Epithelial Cell Culture

The Caco-2 human colon carcinoma epithelial cell line was purchased from the American Type Culture Collection (ATCC, Virginia, USA) and cultured in Eagle's minimum essential medium (Biosera, France) supplemented with 20% fetal bovine serum (Corning, New York, USA) and 1% nonessential amino acid solution (Sigma-Aldrich, Missouri, USA). Cells were maintained in a humidified incubator containing 5% CO_2_ at 37° C.

### 2.8. RNA Isolation and qPCR

Total RNA was extracted from the Caco-2 monolayer using NucleoSpin RNA Plus kit (Macherey-Nagel, Germany) according to the manufacturer's protocol. It was quantified using a Nanodrop spectrophotometer and Qubit 2.0 fluorometer (Thermo Fisher Scientific, USA). 1 *μ*g of total RNA was reverse-transcribed with M-MuLV RT (Maxima First-Strand cDNA Synthesis Kit, Thermo Fisher Scientific, USA). 100 ng cDNA was used in 20 *μ*l reactions for real-time PCR using the Xceed qPCR SG 2× Mix (Institute of Applied Biotechnologies, Praha-Strašnice, Czech Republic) and a CFX96 Touch Real-Time PCR Detection System (Bio-Rad, USA). After 40 cycles of PCR reaction, products were run on a 1.5% agarose gel using 20 bp DNA Ladder (Lonza, Basel, Switzerland). Data were analyzed by *Δ*Ct method. As a reference for gene expression, we used *β*-actin expression. Primers for the investigated gene expression were as follows: (i) *β*-actin (121 bp): forward 5′-GCATGGGTCAGAAGGATTCC-3′, reverse 5′-CAGATTTTCTCCATGTCGTCCC-3′; (ii) PARP-1 (109 bp): forward 5′-CGAGTCGAGTACGCCAAGAG-3′, reverse 5′-CATCAAACATGGGCGACTGC-3′; (iii) PARP-2 (97 bp): forward 5′-GCCAGCAAAAGGGTCTCTGA-3′, reverse 5′-CATGAGCCTTCCCCACCTTG-3′; and (iv) PARP-3 (115 bp): forward 5′-CCTGAGGCTCATGGAGAGTTG-3′, reverse 5′-TGGAGCCATGGCCAAGAAAA-3′. The efficiency of the reactions was in all cases near 100%.

### 2.9. Impedance-Based Barrier Integrity Measurements

First, the epithelial barrier integrity was determined by measuring electrical impedance using xCELLigence RTCA DP Real-Time Cell Analyzer (ACEA Biosciences, California, USA). Caco-2 cells were seeded on RTCA E-plates (E-plate 16) at a density of 10^5^ cells/well. We applied the control (CTRL) and H_2_O_2_ or H_2_O_2_+olaparib treatment groups. After attaining confluency, the monolayers were treated with different concentrations of H_2_O_2_ (100, 200, 500, and 1000 *μ*M) or with olaparib (10 *μ*M) as a pretreatment, 30 min before H_2_O_2_. The CTRL and H_2_O_2_ treatment groups received the same amount of DMSO as the olaparib-treated cells. The cell index (CI) was continuously monitored by the equipment for 24 hours.

### 2.10. FITC-Dextran Epithelial Permeability Assay

Permeability was assessed by measuring the flux of FITC-dextran from the upper compartment to the lower compartment in Transwell plates (pore size 0.4 *μ*m; polyester membrane, Corning, New York, USA). Caco-2 cells were grown until full confluency in 12 well Transwell plates. Here, we applied the same treatment groups as described in the impedance-based technique. Cells were treated with 1000 *μ*M H_2_O_2_ or pretreated with 10 *μ*M olaparib for 30 minutes. After 24 hours, FITC-dextran solution (1 mg/ml) was added to the upper chamber. 1 hour later, a medium from the lower chamber was collected and the fluorescence intensities were detected by a Promega GloMax plate reader (Promega, USA) at 490- nm excitation and 510–570 nm emission wavelengths.

### 2.11. Determination of Apoptosis

Mouse Annexin V & Dead Cell Kit (Merck Millipore, Massachusetts, USA) was used for the quantitative analysis of live, early, and late apoptotic and necrotic cells. Caco-2 cells were seeded onto 6-well plates at a density of 10^6^ cells/well. Treatments and treatment groups were exactly the same as described above at the FITC-dextran assay. 24 hrs after treatment, cells were trypsinized and collected; sample preparation was performed as suggested by the manufacturer. Briefly, 100 *μ*L of cell suspension was incubated with 100 *μ*l of Muse Annexin V & Dead Cell reagent for 20 minutes, in the dark at room temperature. After staining, the assay was performed with a Muse Cell Analyzer (flow cytometer).

### 2.12. Seahorse XFp Cell Mito Stress Test

Measurement of the oxygen consumption rate (OCR) and extracellular acidification rate (ECAR) in Caco-2 monolayers was performed by a Seahorse XFp Extracellular Flux Analyzer (Agilent Technologies, California, USA). The day before the assay, the Seahorse XFp Sensor Cartridge was hydrated with XF Calibrant Solution and was kept at 37°C in a CO_2_-free incubator overnight. Caco-2 cells were seeded on XFp Miniplates at a density of 1.5 × 10^4^ cells/well. After reaching 100% confluence, cells were treated exactly as described at the FITC-dextran assay. After the treatment, a complete growth medium was replaced with an unbuffered, serum-free Agilent XF Base assay medium, pH 7.4. XFp Mito Stress Test Kit was used to test mitochondrial function. Injection of oligomycin, carbonyl cyanide-4 (trifluoromethoxy) phenylhydrazone (FCCP), and the mix of rotenone and antimycin A allows determining the key bioenergetic parameters: basal respiration, ATP production-linked respiration (ATP production), maximal respiration, spare respiratory capacity, nonmitochondrial respiration, proton leak, and coupling efficiency. Oligomycin inhibits the F_O_ subunit of the F_O_F_1_-ATP synthase, thereby indicating ATP-linked OCR, i.e., level of ATP synthesis. ATP-linked respiration is calculated by the difference between baseline OCR and OCR after oligomycin injection. Distracting nonmitochondrial respiration from the OCR after FCCP injection represents maximal respiration. FCCP is a mitochondrial uncoupler, which separates the activity of phosphorylation and oxidation. Under these circumstances, ETC might work with its maximum rate and consumes higher amounts of O_2_ without developing membrane potential between the two sides of the mitochondrial inner membrane. Spare respiratory capacity is defined by the difference between maximal and basal respiration. The mixture of rotenone and antimycin A inhibits CI and CIII, respectively; thus, mitochondrial ETC and O_2_ consumption are blocked. The final concentrations of the modulators were 1 *μ*M. OCR after rotenone/antimycin A injection represents nonmitochondrial respiration. ATP-linked respiration divided by basal respiration reveals coupling efficiency.

### 2.13. Light Microscopy Imaging

Caco-2 cells were seeded at a density of 10^6^ cells/well on 6-well plates. After reaching confluency, the monolayers were treated exactly as described at the FITC-dextran assay. 24 hours later, monolayers were visualized by EVOS XL Core Cell Imaging System (Thermo Fisher Scientific, USA) using a 20× objective.

### 2.14. Statistical Analysis

Experimental data were analyzed by using GraphPad Prism Software (GraphPad Software Inc., California, USA). Statistical difference between groups was established by Student's *t*-test, with Bonferroni correction; *P* values less than 0.05 were considered statistically significant.

## 3. Results

### 3.1. Olaparib Improved TNBS-Colitis in Mice

**Figure 2:**
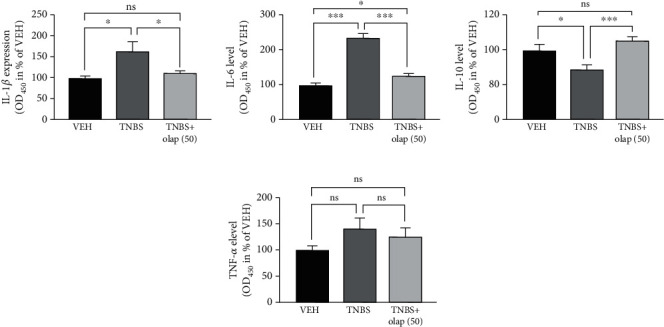
Olaparib decreased colonic proinflammatory cytokine (IL-1*β* and IL-6) and increased anti-inflammatory IL-10 cytokine levels. Cytokine levels of (a) IL-1*β*, (b) IL-6, (c) IL-10, and (d) TNF-*α*. Data combined from 2 separate experiments (*n* = 9-13) and expressed as mean ± SEM; ns: not significant; ^∗^*P* < 0.05, ^∗∗^*P* < 0.01, and ^∗∗∗^*P* < 0.001. VEH: vehicle; TNBS: 2,4,6-trinitrobenzene sulphonic acid; olap (50): 50 mg/kg olaparib.

**Figure 3:**
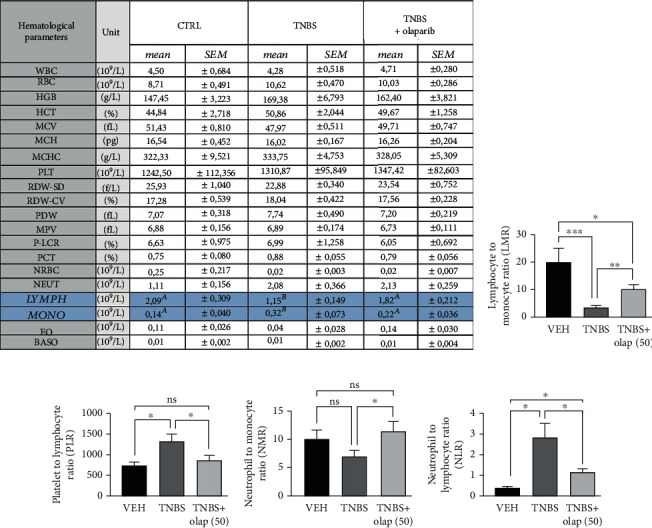
Olaparib modified hematological parameters and improved blood cell ratios in TNBS-treated mice. (a) Hematological parameters in the VEH, TNBS, and TNBS+olap (50) groups. Mean values ± SEM are shown in the table. A, B = different letters in each row indicate significant differences between groups (*P* < 0.05). Data combined from 3 separate experiments (*n* = 9-20). (b) Lymphocyte to monocyte ratio (LMR), (c) platelet to lymphocyte ratio (PLR), (d) neutrophil to monocyte ratio (NMR) (e), and neutrophil to lymphocyte ratio (NLR) values were calculated for each mouse individually before the averages were determined. Mean ± SEM is shown; ns: not significant; ^∗^*P* < 0.05, ^∗∗^*P* < 0.01, and ^∗∗∗^*P* < 0.001. Data are combined from 3 separate experiments (*n* = 9-20). Abbreviations: WBC: white blood cell number; RBC: red blood cell number; HGB: hemoglobin; HCT: hematocrit; MCV: mean corpuscular volume; MCH: mean corpuscular hemoglobin; MCHC: mean corpuscular hemoglobin concentration; PLT: platelet count; RDW-SD: red cell distribution width-standard deviation; RDW-CV: red cell distribution width-coefficient of variation; PDW: platelet distribution width; MPV: mean platelet volume; P-LCR: platelet large cell ratio; PCT: procalcitonin; NRBC: nucleated red blood cells; NEUT: neutrophil count; LYMPH: lymphocyte count; MONO: monocyte count; EO: eosinophil count; BASO: basophil count.

To evaluate the effect of olaparib in experimental colitis, we used the TNBS-colitis model (Figure 1(a)), a mouse model of CD [35]. Olaparib was used as a pretreatment in 20 and 50 mg/kg bodyweight dose. On the one hand, olaparib failed to significantly ameliorate weight loss in TNBS-challenged animals (Figure 1(b)). But on the other hand, it decreased inflammation scores by more than ~50% in 50 mg/kg (*n* = 21), but not in 20 mg/kg dosage (*n* = 9) (Figure 1(d)). Hence, we used 50 mg/kg dose in the further experiments. Olaparib impeded histological injury in the colon (Figure 1(c)), reduced the number of ulcers (*n* = 27) (Figure 1(e)) and their lengths (*n* = 27) (Figure 1(f)), and most importantly, diminished FITC-dextran permeability (*n* = 8) (Figure 1(g)) compared to the CTRL group (*n* = 5). Levels of inflammatory cytokines were also modulated. Olaparib diminished IL-1*β* (Figure 2(a)) and IL-6 (Figure 2(b)) proinflammatory cytokine levels, but enhanced anti-inflammatory IL-10 production (Figure 2(c)) in the colon (*n* = 13). Interestingly, we could not find statistically significant alteration in the TNF-*α* level (Figure 2(a)). We also evaluated numerous hematological parameters in colitic mice (Figure 3(a)). We found only 2 parameters, namely, the amounts of lymphocytes and monocytes, which were significantly modulated by the treatments. In agreement with others' findings on colitis models, TNBS substantially reduced lymphocyte number in mice (*n* = 9), while olaparib counteracted this effect (*n* = 20). In contrast, monocyte number was higher in the TNBS group (*n* = 9), whereas it was significantly less elevated in the TNBS+olaparib group (Figure 3(a)) (*n* = 20). We calculated specific blood cell ratios, which were previously shown to be changed in CD [36] based upon the individual blood cell counts. Similarly to CD, neutrophil to lymphocyte ratio (NLR) (Figure 3(e)) and platelet to lymphocyte ratio (PLR) (Figure 3(c)) were both increased in TNBS-colitis and they were markedly reduced by olaparib treatment. Again, as in CD, lymphocyte to monocyte ratio (LMR) (Figure 3(b)) was reduced in experimental colitis, and it was amended by the PARP inhibitor. Unfortunately, TNBS-induced changes in the neutrophil to monocyte ratio (NMR) (Figure 3(d)) did not reach statistical significance compared to the vehicle. However, olaparib improved NMR related to the TNBS-treated group.

### 3.2. Caco-2 Colonic Epithelial Cells Expressed PARP-1, PARP-2 and PARP-3

**Figure 4:**
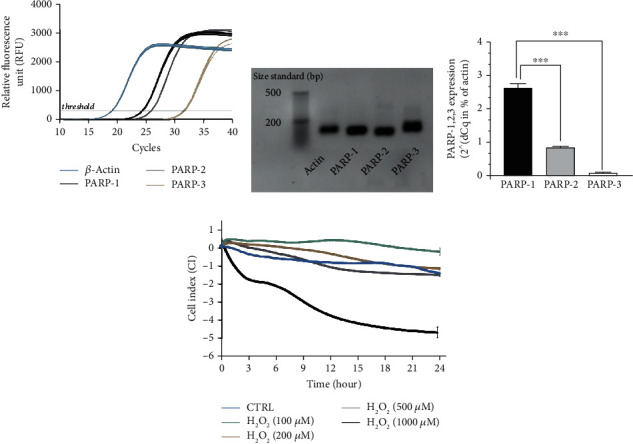
PARP-1, PARP-2, and PARP-3 are expressed in Caco-2 monolayers and high concentration of H_2_O_2_ disrupted barrier integrity. (a) Representative amplification curves of PARP-1, PARP-2, PARP-3, and *β*-actin quantitative real-time PCR (each containing three biological replicates and three technical replicates (data for biological replicates is not shown)). *β*-Actin is used as reference gene. (b) Agarose gel electrophoresis of the PCR products of the amplification (after 40th cycles) introduced in (a). Nonquantitative gene amplicons visualized in a 1.5% agarose gel. (product size: *β*-actin: 121 bp, PARP-1: 109 bp, PARP-2: 97 bp, and PARP-3: 115 bp; size standard from LONZA DNA ladder, 20 bp). (c) Relative gene expression results (2^(-dCq mean)), where *β*-actin expression is considered 100% (data is not shown). The relative differences (dCq) between the average Cq for the *β*-actin and the mean Cq per individual samples (PARP-1, PARP-2, and PARP-3). (d) Effect of H_2_O_2_ at different concentrations (100-1000 *μ*M) on the disruption of epithelial barrier integrity of Caco-2 monolayers. Electrical impedance was monitored every 3 minutes for 24 hours with an xCELLigence RTCA instrument. Impedance-related cell index (CI) values from one of three independent experiments are expressed as mean ± SD (*n* = 4-6).

Olaparib has been shown to inhibit three members of the PARP enzyme family, namely, PARP-1 (IC_50_ = 5 nM), PARP-2 (IC_50_ = 1 nM), and PARP-3 (IC_50_ = 4 nM) [37]. Thus, we investigated the basal expression profile of the three target isoforms in untreated Caco-2 cells forming a confluent monolayer. We detected continuous PARP-1, PARP-2, and PARP-3 mRNA expressions (Figures 4(a)–4(c) ), but with different expression rates (PARP-1 > PARP-2 > PARP-3) (Figure 4(c)). In Caco-2 cells, PARP-1 was the most highly expressed isoform. PARP-2 and PARP-3 mRNA expressions were at about ~9-fold and~335-fold weaker compared to PARP-1 (Figure 4(c)).

### 3.3. Olaparib Improved Barrier Function of Epithelial Monolayer in Oxidative Stress

**Figure 5:**
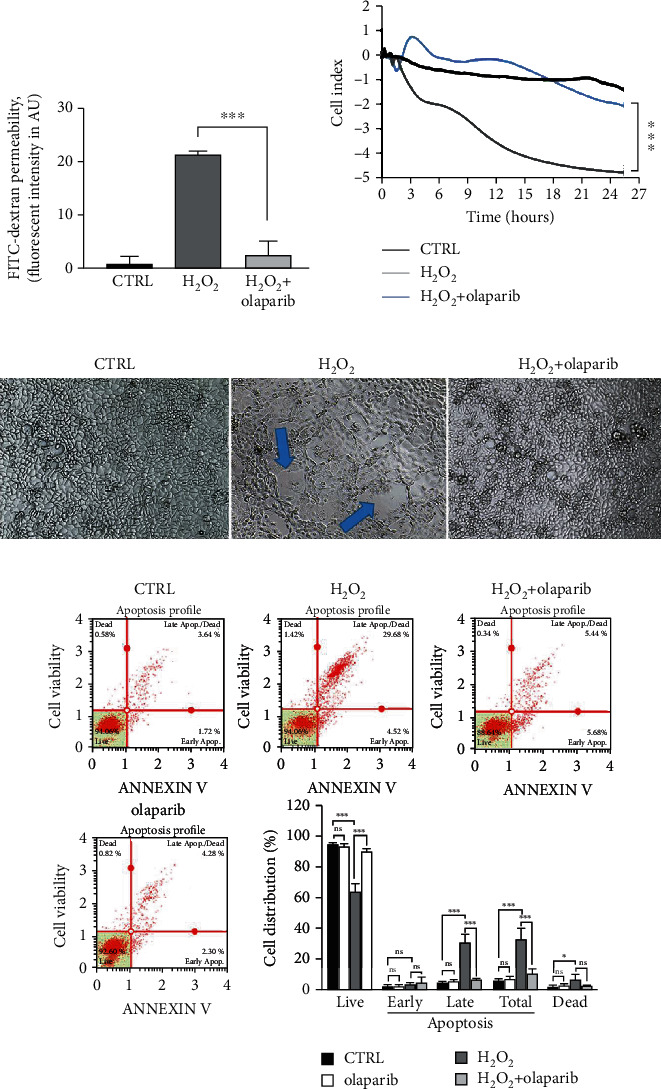
Olaparib improved epithelial barrier integrity in H_2_O_2_-treated Caco-2 monolayers. Caco-2 monolayers were pretreated with 10 *μ*M olaparib for 30 minutes before 1 mM H_2_O_2_ treatment for 24 hours in all experimental settings. The CTRL and H_2_O_2_ groups received the same amount of DMSO as the olaparib-treated cells. (a) FITC-dextran epithelial permeability assay. 40 kDa FITC-dextran (1 mg/ml) was added to the upper chamber of the Transwell plate. After 1 hour of incubation, the medium from the lower chamber was collected and the fluorescence intensities were measured. Data combined from 2 separate experiments (*n* = 4). (b) Electrical impedance monitoring of Caco-2 monolayers using E-Plates and xCELLigence RTCA instrument. Data from one of three experiments are expressed as mean ± SD (*n* = 4-6). (c) Representative light microscopy images of confluent Caco-2 monolayers after 24 hours treatment. (d) Determination of apoptosis and necrosis in Caco-2 monolayers analyzed by Muse Annexin V & Dead Cell Kit. Representative apoptosis profiles and the percentage of live, early apoptotic, late apoptotic, total apoptotic, and necrotic cells are presented. Data are combined from 2 separate experiments (*n* = 4) and expressed as mean ± SD; ns: not significant; ^∗^*P* < 0.05, ^∗∗^*P* < 0.01, and ^∗∗∗^*P* < 0.001.

Caco-2 monolayers are widely used as a model for intestinal epithelial barrier [38]. Since the activity of PARP-1, PARP-2, and PARP-3 isoforms can be induced by DNA-damage [39], and as oxidative stress induces mucosal injury in IBD [40, 41], we tested different H_2_O_2_ concentrations (100-1000 *μ*M) on Caco-2 monolayers. We assessed barrier integrity by an impedance-based technique (Figure 4(d)). Lower concentrations of H_2_O_2_ (100-500 *μ*M) did not considerably modify cell index (CI; calculated from TEER impedance values) meaning they did not impair barrier integrity. However, 1 mM H_2_O_2_ rapidly and permanently decreased CI. After 24 hrs, 1 mM H_2_O_2_ strongly eroded the epithelial monolayer (Figure 4(d)). Consequently, in the further experiments, we applied 1 mM concentration of H_2_O_2_ to challenge the barrier. Olaparib pretreatment, 30 min before H_2_O_2_ exposure, improved CI compared to H_2_O_2_-treated cells and protected monolayer integrity (Figure 5(b)). To confirm these findings, we also performed FITC-dextran *trans*-epithelial permeability assay in the same model at the endpoint of the impedance-based measurement, after 24 hrs incubation (Figure 5(a)). We detected about ~20-fold increase in FITC-dextran fluorescent intensity after H_2_O_2_ treatment (i.e., FITC-dextran could pass the monolayer) in relation to CTRL. In contrast, olaparib reduced H_2_O_2_-induced FITC-dextran permeability near to the level of control (Figure 5(a)). To even further refine our results, we performed microscopic imaging and observed morphological changes in the structure of epithelial monolayer after H_2_O_2_ treatment. We realized compromised, in some places broken monolayer, with presumably dying cells, which lost their connection to neighbors in the monolayer. Olaparib prevented these morphological changes and kept the cells as an integral part of the barrier in their normal, epithelial phenotype (Figure 5(c)).

### 3.4. Olaparib Protected against Oxidative Stress-Induced Cell Death in Epithelial Barrier

To assess whether oxidative stress-induced barrier dysfunction involves epithelial cell death we performed flow cytometry analysis using Annexin V/7-AAD labeling (Figure 5(d)). H_2_O_2_ (1 mM) induced a marked increase in the amount of 7-AAD positive dead, basically necrotic cells (5.87% of total cells; 4.89-fold increase) compared to CTRL. In addition, it enhanced the annexin V/7-AAD double-positive, late apoptotic cell number (31.9% of total cells; 6.86-fold increase). In our hands, H_2_O_2_ had no significant effect on early apoptosis. Olaparib protected against H_2_O_2_-induced cell death, i.e., it reduced necrotic cell death (1.25% of total cells; 4.70-fold decrease) and late apoptosis (5.73%; 5.17-fold decrease) almost to the level of CTRL (Figure 5(d)).

### 3.5. PARP Inhibition Recovered Glycolytic Activity Compromised by H_2_O_2_ Treatment

**Figure 6:**
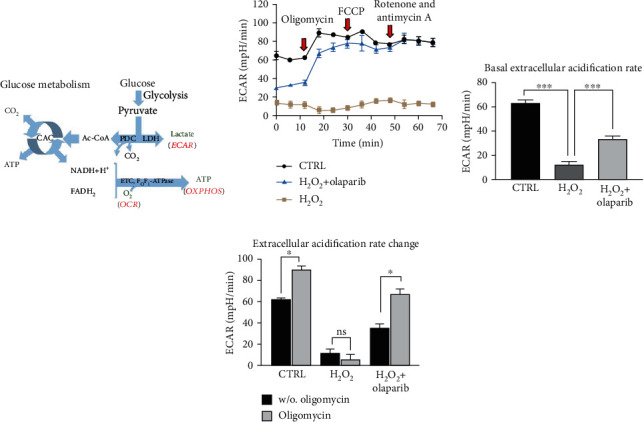
Olaparib improved aerobic glycolysis in Caco-2 monolayers exposed to H_2_O_2_-induced oxidative stress. Seahorse XFp Mito Stress test was performed after Caco-2 monolayers were pretreated with 10 *μ*M olaparib for 30 minutes and then treated with 1000 *μ*M H_2_O_2_ for 2 hours. The CTRL and H_2_O_2_ groups received the same amount of DMSO as the olaparib-treated cells. Oligomycin (1 *μ*M), FCCP (1 *μ*M), and the mixture of rotenone and antimycin A (1 *μ*M) were added sequentially during the measurement. (a) Schematic illustration of glucose metabolism. (b) Extracellular acidification rate (ECAR), (c) basal ECAR, and (d) ECAR changes are shown. ECAR changes were calculated by the difference between ECAR before and after oligomycin injection. Data combined from 2 separate experiments (*n* = 4); ns: not significant; ^∗^*P* < 0.05, ^∗∗^*P* < 0.01, and ^∗∗∗^*P* < 0.001. Abbreviations: PDC: pyruvate dehydrogenase complex; LDH: lactate dehydrogenase; CAC: citric acid cycle; ETC: mitochondrial electron transport chain; ECAR: extracellular acidification rate; OCR: oxygen consumption rate; OXPHOS: oxidative phosphorylation.

In inflammation, colonocytes switch their oxidative metabolism (butyrate consumption) to aerobic glycolysis and produce lactate [13, 17] (Figure 6(a)). Thus, we investigated the glycolytic activity by measuring extracellular acidification rate (ECAR), i.e., lactate production (Figure 6(b)), two hours after H_2_O_2_ treatment in the early phase of oxidative stress. H_2_O_2_ caused a dramatic collapse in basal ECAR (w/o oligomycin) compared to CTRL, which was markedly enhanced by olaparib (Figure 6(b) (1-3 points of the measurement) and Figure 6(c)). Oligomycin treatment increased ECAR both in the CTRL and H_2_O_2_+olaparib-treated cells compared to the untreated (w/o oligomycin) group but failed to stimulate acidification in the H_2_O_2_-damaged monolayer (Figure 6(b) (4-6 points of measurement) and Figure 6(d)). FCCP, rotenone, and antimycin A did not influence ECAR significantly in either treatment groups (Figure 6(b) (7-12 points of measurement)).

### 3.6. PARP Inhibitor Olaparib Preserved Mitochondrial Respiration in H_2_O_2_-Induced Stress

**Figure 7:**
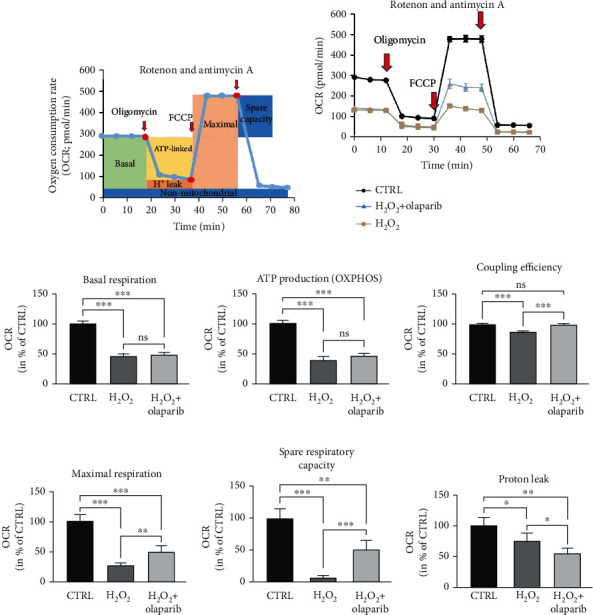
Olaparib protected mitochondrial function in Caco-2 monolayers subjected to H_2_O_2_-induced oxidative stress. Caco-2 monolayers were pretreated with 10 *μ*M olaparib for 30 minutes and then treated with 1000 *μ*M H_2_O_2_ for 2 hours. The CTRL and H_2_O_2_ groups received the same amount of DMSO as the olaparib-treated cells. Seahorse XFp Mito Stress test was performed, when oligomycin (1 *μ*M), FCCP (1 *μ*M), and the mixture of rotenone and antimycin A (1 *μ*M) were injected sequentially. (a) Key parameters of mitochondrial respiration measured by Seahorse XFp Extracellular Flux Analyzer. (b) Measurement of oxygen consumption rate (OCR). Data combined from 2 separate experiments are expressed as mean ± SD (*n* = 4). Bioenergetic parameters: (c) basal respiration, (d) ATP production, (e) coupling efficiency, (f) maximal respiration, (g) spare respiratory capacity, and (h) proton leak were calculated. Results are expressed in percentage of control (mean ± SD of two independent experiments, *n* = 4); ns: not significant; ^∗∗^*P* < 0.01, ^∗∗∗^*P* < 0.001.

Under physiologic conditions, butyrate is the main source of ATP in colonocytes [12], and butyrate metabolism involves dynamic mitochondrial ETC activity and continuous OXPHOS [42]. Therefore, we investigated the activity of ETC and OXPHOS by measuring the oxygen consumption rate in our epithelial barrier model (Figure 7(a)). First, the basal respiration (OCR w/o oligomycin, green field on Figure 7(a)) was determined. H_2_O_2_ reduced basal respiration in epithelial cells compared to CTRL, and olaparib did not modulate this effect (Figure 7(b) (1-3 points of measurement and Figure 7(c)) indicating that olaparib had no effect on basal respiration. After oligomycin treatment, the ATP production-linked OCR (OCR with oligomycin, yellow field on Figure 7(a)) can be measured that reflects the activity of OXPHOS and ATP generation. Oligomycin reduced OCR and OXPHOS xoverall in all three experimental groups (Figure 7(b) (4-6 points of measurement]) and Figure 7(d)), but H_2_O_2_-treated cells consumed O_2_ even to a lesser extent than CTRL, which suggested a reduced ATP production. Olaparib had no significant effect on the ATP-linked OCR in H_2_O_2_-treated cells (Figure 7(d)). Furthermore, olaparib ameliorated the H_2_O_2_-induced decline in coupling efficiency (Figure 7(e)). In contrast, FCCP, an uncoupling agent that induces maximal respiration in the mitochondria (OCR with FCCP, beige field on Figure 7(a)), enhanced OCR in all three groups in different extents (Figure 7(b) (7-10 points of measurement)). We detected the highest OCR in CTRL, the lowest in the H_2_O_2_-induced cells while olaparib counteracted the effect of H_2_O_2_ (Figure 7(b) (7-10 points of measurement) and Figure 7(f)). FCCP application also determined spare respiratory capacity (blue field on Figure 7(a)). Spare respiratory capacity was intensely reduced by H_2_O_2_ compared to CTRL, but olaparib attenuated this reduction (Figure 7(g)). H_2_O_2_ reduced proton leak (orange field on Figure 7(a)) compared to CTRL, and it was further reduced by olaparib in Caco-2 cells (Figure 7(h)).

## 4. Discussion

The anti-inflammatory role of PARP inhibition is thoroughly established; however, introduction of PARP inhibitors into clinical therapy of anti-inflammatory diseases has not been initiated yet because of the potential risk in long-term use of the drugs [33]. In this study, we intended to provide experimental support for repositioning the PARP inhibitors (which are successfully applied in human cancer therapy) for the clinical management of the acute flare-up periods of CD. For that purpose, we used a TNBS-induced experimental colitis model in mice, which is widely accepted for studying CD, since they share many pathological (clinical, histological, and biochemical) characteristics [35]. Furthermore, enhanced PARP-1 expression was found in the colon of rodents [43, 44] in experimental colitis models, as well as in IBD patients [45], which makes the model more valuable for studying PARP inhibitors. In this report, we explicitly focused on the epithelial barrier function, cell survival, and bioenergetics; hence, we used a Caco-2 monolayer as an *in vitro* model of intestinal epithelial barrier [46]. We demonstrate that olaparib improves inflammation in TNBS-colitic mice and that it protects Caco-2 epithelial barrier in oxidative stress by rescuing glycolytic activity and by protecting some aspects of mitochondrial function.

In a recent review about repurposing PARP inhibitors for the therapy of nononcological diseases [33], Berger et al. did not consider IBD among those chronic diseases, in which the assumed benefits vs. the risks justify first priority of repurposing. However, the available preclinical data on IBD models successfully utilized outdated PARP inhibitors such as 3-aminobenzamide [47]. Our findings of *in vivo* anti-inflammatory effects of olaparib, a PARP inhibitor approved for human cancer therapy, may justify initiation of clinical trials for repurposing this drug for IBD therapy. We hypothesize that PARP inhibition might be beneficial in the acute flare-ups of severe CD, where detrimental ulceration and tissue damage are caused by the energetic collapse of mucosal cells. It could be especially true in the severe cases of drug nonresponders, e.g., those one-third of IBD patients who primarily do not respond to infliximab (anti-TNF-*α* mAb; commonly prescribed drug in IBD) [48] or to other pharmacological therapies.

We demonstrated that olaparib improved TNBS-induced colitis in mice, reduced histological damage of the colon, diminished the number and length of the ulcers, inhibited proinflammatory cytokine production (IL-1*β*, IL-6), but it enhanced the level of anti-inflammatory cytokine IL-10. Inflammatory cytokines participating in the generation of colon damage are predominantly produced by activated leukocytes [49]. The fact that PARP inhibition reduces leukocyte infiltration into the colon in experimental colitis is well characterized [50–52]. Accordingly, we investigated hematological parameters from peripheral blood in our TNBS-colitis model. Several types of blood cell ratios were recently highlighted as possible diagnostic parameters in IBD [36, 53]. Specifically in CD, NLR and PLR were suggested to be valuable diagnostic factors [36]. Moreover, NLR might predict disease severity [54]; however, this notion is debated [55]. In detail, elevated NLR, PLR, and reduced LMR were found in CD patients compared to control subjects, while NMR was not modified [36]. In our experiments, alterations in NLR, PLR, LMR ,and NMR in TNBS-treated mice followed the observed changes in CD patients. In addition, olaparib effectively reversed CD-specific alterations in PLR and LMR, and most importantly, it reduced NLR. NLR was found to be a significant predictor of infliximab drug response in CD patients [56]. That is, the anti-inflammatory efficacy of infliximab correlated with decreased NLR in CD. Olaparib's identical anti-inflammatory effect in our TNBS-colitis model underlines the drug's potential in CD therapy.

Elevated NLR can also refer to oxidative stress in CD patients [57], which is an important inducer of PARP activation [58]. In active CD, excessive amounts of ROS are produced by the immune cells. The main source of ROS in immune cells is the H_2_O_2_ production [59], and H_2_O_2_ causes DNA damage in colonocytes [60] that triggers PARP activation. High concentrations of ROS result in apoptosis of IECs leading to disruption of epithelial barrier integrity in the colon, which is a definite hallmark of IBD. Accordingly, we treated Caco-2 monolayer with high concentration of H_2_O_2_ (1 mM) to imitate a strong oxidative stress-injured barrier, *in vitro*. We demonstrated that Caco-2 monolayer cells expressed PARP-1 mRNA in a high extent similarly to colonic mucosa [45]. In addition, we detected PARP-2 and PARP-3 expressions, however, in a decreasingly lower extent. That is, Caco-2 cells express the mRNA of olaparib's target enzymes (PARP-1, PARP-2, and PARP-3); furthermore, in the monolayer, these cells mimic the intestinal barrier [46]. Therefore, the Caco-2 monolayer seemed to be an appropriate model to investigate the effect of PARP-inhibition on barrier integrity, *in vitro*. Our results showed that olaparib preserved the Caco-2 monolayer integrity in oxidative stress and protected the epithelial cells from apoptosis. These findings indicated that colonic epithelial cells might be direct targets of olaparib in TNBS-colitis, and barrier protection might be one of the key components of its anti-inflammatory action.

**Figure 8:**
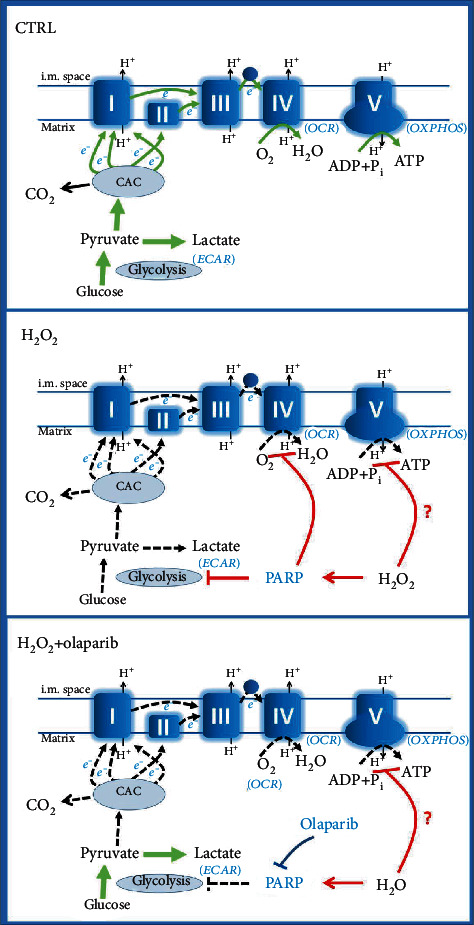
Schematic illustration of the assumed mechanistic effect of olaparib. CAC: citric acid cycle; OCR: oxygen consumption rate; OXPHOS: oxidative phosphorylation; ECAR: extracellular acidification rate; PARP: poly(ADP-ribose) polymerase.

Reduced barrier integrity and increased gut permeability are typical signs of IBD. They have been recently associated with epithelial cell death, mitochondrial dysfunction, and depleted energy metabolism in IECs. IECs produce ATP predominantly by aerobic glycolysis in colitis. However, overactivation of PARP might fully block glycolysis [25]. To study the metabolic effect of olaparib in epithelial cell death, we induced powerful oxidative stress (1 mM H_2_O_2_, 2 hrs) in Caco-2 monolayer and determined various parameters of the energy metabolism (Figure 8). The H_2_O_2_ stress dramatically decreased basal ECAR (basal glycolysis) and induced energetic collapse in Caco-2 cells. Lower proton leak rate in H_2_O_2_-treated cells compared to control also reflected this metabolic failure, because ATP demand reduces proton motive force and diminish proton leakage [61, 62]. In contrast, olaparib significantly enhanced ECAR in H_2_O_2_-treated cells. Our results are strongly supported by the finding that poly(ADP-ribose) (PAR) binds to the PAR-binding motif in hexokinase and inhibits it thereby reducing glycolysis [25]. These data indicate that olaparib exerted its effect by inhibiting PAR production, thereby preventing PAR-mediated inhibition of hexokinase and the blockade of aerobic glycolysis (Figure 8).

Oligomycin is a F_O_F_1_-ATPase inhibitor, which blocks OXPHOS. As expected, oligomycin had no effect on ECAR in the H_2_O_2_ treatment group; however, it intensely increased ECAR in H_2_O_2_+olaparib-treated cells. An explanation for this finding might be that oligomycin blocked ATP synthesis and cells could not compensate for the lack of ATP by boosting glycolysis in H_2_O_2_ treatment group because of the strong PARP-mediated repression of hexokinase. Olaparib, however, prevented PARP activation and glycolytic collapse in H_2_O_2_+olaparib-treated cells (Figure 8) even in the presence of oligomycin. One may say that oxidative stress per se might affect glycolytic enzymes and not only PARP activation regulates glycolytic activity. By using FCCP (mitochondrial uncoupler), rotenone, and antimycin A (inhibitors of ETC) mitochondrial energy production is totally abrogated. Under these circumstances, glycolysis remains the ultimate source of ATP (Figure 8). The fact that ECAR could reach the level of CTRL cells in the H_2_O_2_+olaparib group after FCCP, rotenone and antimycin A treatment clearly indicated that glycolytic enzymes were not significantly affected by the oxidative stress in our system, and glycolytic energy production was controlled by PARP activation. Thus, olaparib protected from PARP-induced energetic collapse by improving aerobic glycolysis in oxidative stress (Figure 8).

Previous studies indicated that the mitochondria are the primary source of ROS in IECs during inflammation [63] and PARP-1 activation in oxidative stress causes mitochondrial dysfunction [25]. Accordingly, we wanted to know whether olaparib can prevent mitochondrial failure in our model. We found that basal respiration, mitochondrial ATP production, and nonmitochondrial oxygen consumption were strongly impeded in oxidative stress, and olaparib could not reverse these changes. However, it increased maximal respiration, spare respiratory capacity, and coupling efficiency and reduced proton leak. For understanding these results, we should consider the direct and indirect effects of H_2_O_2_ on CAC enzymes, ETC complexes, and F_O_F_1_-ATPase and the effects of PARP activation on glycolysis and ETC complexes.

Previous studies using the DNA-alkylating agent N-methyl-N-nitroso-N-nitroguanidine (MNNG) for PARP activation reported mitochondrial dysfunction [25]. MNNG treatment resulted in decreased basal OCR, maximal OCR, ATP synthesis, and ECAR, while PARP inhibition significantly reversed these changes. Most importantly, they found that administration of pyruvate completely prevented MNNG-induced mitochondrial failure [25]. That is, the mitochondrial dysfunction was a direct consequence of downregulated glycolysis, i.e., it compromised fuel supply for the CAC and ETC. Our results led to the same conclusion, namely, PARP activation reduced glycolysis and caused mitochondrial dysfunction (Figure 8).

In contrast to MNNG-induced PARP activation, we found that inhibition of the enzyme did not prevent the H_2_O_2_-induced reduction of basal respiration and mitochondrial ATP production. On the other hand, olaparib enhanced ECAR, i.e., it effectively prevented glycolytic collapse (Figure 8). Additionally, it increased maximal respiration, spare respiratory capacity, coupling efficiency, and reduced proton leak, i.e., it increased efficacy of OXPHOS. The discrepancy between our results and the previous ones [25] can be resolved by considering that MNNG alkylates the DNA leading to DNA breaks and PARP activation. H_2_O_2_, however, induces oxidative DNA damage-mediated PARP activation but can cause direct structural impairment to CAC, ETC, and F_O_F_1_-ATPase components as collateral damage. It is well established that H_2_O_2_ deteriorates CAC activity and reduces proton motive force, which is a prerequisite for mitochondrial pyruvate transport [64]. Also, F_O_F_1_-ATPase was reported to be susceptible to oxidative stress [65, 66]. In addition, ROS and especially H_2_O_2_ can directly block many components of the respiratory chain, such as NADH dehydrogenase or cytochrome c oxidase [65]. Our findings that maximal OCR of the H_2_O_2_+olaparib treatment group could not reach the maximum OCR level of control cells are in line with the notion that ETC is sensitive toward oxidative stress (Figure 8). The observed difference between maximal OCRs of the two groups could be the result of oxidative damage to CAC, ETC, and F_O_F_1_-ATPase components in the H_2_O_2_+olaparib group. Because the glycolytic fuel supply pathway was unimpeded in both groups, thanks to olaparib's inhibitory effect on PARP in the H_2_O_2_+olaparib group. Furthermore, the observed increase of maximal OCR in the H_2_O_2_+olaparib group vs. the H_2_O_2_ group could result from direct control of the ETC by PARylation. Studies found excessively PARylated mitochondrial proteins, including components of ETC. In addition, PARP-inhibitors such as 3-aminobenzamide and nicotinamide prevented the H_2_O_2_-induced electron transport blockade on CIV in isolated mitochondria [67]. These results suggest that PARP could regulate ETC activity on CIV, and it also proposed a mitochondrial target for PARP inhibitors. Another study suggested a pivotal role for PARP-1 in mitochondrial energy homeostasis and demonstrated CI as a mitochondrial target of PARP-1 activation [68]. However, it should be noted that the existence of PARP-1 or other PARP isoforms in the mitochondria is debated. But whether present or not in the mitochondria, PARP has a clear effect on mitochondrial function [69].

## 5. Conclusion

In conclusion, olaparib, a PARP inhibitor used in human oncotherapy, restored bioenergetics by glycolytic reactivation of colonic epithelial cells, and it decreased cell death. Epithelial cell protection might be a cause of improved barrier function that eventually resulted in reduced incidence and severity of CD-like symptoms in an experimental rodent IBD model. However these findings provide experimental evidence for repurposing olaparib for IBD treatment and highlight its potential in the therapy of CD; clinical application of the drug in IBD needs further investigations.

## Data Availability

The data underlying this article are available in the article.
